# Supplementary material for: Multi‐dataset identification of innovative feature genes and molecular mechanisms in keratoconus
Source: J Cell Mol Med. 2024 Sep 19;28(18):e70079. doi: 10.1111/jcmm.70079 (PMC11412914; doi:10.1111/jcmm.70079)
Supplement: Supplementary file 2 — Material S2. [file JCMM-28-e70079-s002.zip › 7.GSEA analysis/3.DBNDD1/KEGG_PHENYLALANINE_METABOLISM.html]

Details for gene set KEGG\_PHENYLALANINE\_METABOLISM[GSEA]

|  || Dataset | DBNDD1.group.cls#h\_versus\_l |
| Phenotype | group.cls#h\_versus\_l |
| Upregulated in class | h |
| GeneSet | KEGG\_PHENYLALANINE\_METABOLISM |
| Enrichment Score (ES) | 0.36622733 |
| Normalized Enrichment Score (NES) | 1.0105944 |
| Nominal p-value | 0.45731708 |
| FDR q-value | 1.0 |
| FWER p-Value | 1.0 |
Table: GSEA Results Summary

  

Fig 1: Enrichment plot: KEGG\_PHENYLALANINE\_METABOLISM      
 Profile of the Running ES Score & Positions of GeneSet Members on the Rank Ordered List

  

| SYMBOL | TITLE | RANK IN GENE LIST | RANK METRIC SCORE | RUNNING ES | CORE ENRICHMENT || 1 | MAOB | na | 696 | 0.415 | 0.1446 | Yes |
| 2 | TAT | na | 2033 | 0.298 | 0.2244 | Yes |
| 3 | IL4I1 | na | 3376 | 0.237 | 0.2796 | Yes |
| 4 | AOC2 | na | 4742 | 0.188 | 0.3151 | Yes |
| 5 | PAH | na | 5309 | 0.170 | 0.3662 | Yes |
| 6 | ALDH3B1 | na | 8402 | 0.096 | 0.3153 | No |
| 7 | GOT2 | na | 13748 | 0.032 | 0.1741 | No |
| 8 | DDC | na | 16986 | 0.009 | 0.0846 | No |
| 9 | MAOA | na | 19119 | -0.005 | 0.0253 | No |
| 10 | MIF | na | 19184 | -0.006 | 0.0258 | No |
| 11 | GOT1 | na | 19996 | -0.013 | 0.0076 | No |
| 12 | PRDX6 | na | 20245 | -0.015 | 0.0065 | No |
| 13 | ALDH3A1 | na | 23496 | -0.049 | -0.0675 | No |
| 14 | ALDH3B2 | na | 28095 | -0.133 | -0.1470 | No |
| 15 | HPD | na | 29676 | -0.178 | -0.1218 | No |
| 16 | ALDH1A3 | na | 31867 | -0.257 | -0.0829 | No |
| 17 | AOC3 | na | 33896 | -0.416 | 0.0240 | No |
Table: GSEA details [plain text format]

  

Fig 2: KEGG\_PHENYLALANINE\_METABOLISM      
 Blue-Pink O' Gram in the Space of the Analyzed GeneSet

  

Fig 3: KEGG\_PHENYLALANINE\_METABOLISM: Random ES distribution      
 Gene set null distribution of ES for **KEGG\_PHENYLALANINE\_METABOLISM**
